# [Corrigendum] Effects of histone deacetylase inhibitors on ATP‑binding cassette transporters in lung cancer A549 and colorectal cancer HCT116 cells

**DOI:** 10.3892/ol.2023.14084

**Published:** 2023-10-02

**Authors:** Hao Wang, Chun-Hua Chi, Ying Zhang, Bin Shi, Ru Jia, Ben-Jun Wang

Oncol Lett 18: 63–71, 2019; DOI: 10.3892/ol.2019.10319

Following the publication of this article, the authors drew to the Editor's attention that errors had been made in terms of the compilation and assembly of a pair of the figures. First, the α-tubulin bands featured in Fig. 3B on p. 68 were inadvertently included as the control western blots for the HCT116 cell line in Fig. 2B on p. 67. Secondly, the ‘SAHA’ and ‘TSA’ protein bands featured for the ABCC1 experiment were inadvertently copied across for the ‘DMSO’ and ‘SAHA’ bands for the ABCC5 experiment with the HCT116 cell line in Fig. 2B. Lastly, the α-tubulin bands included in Fig. 4 on p. 69 had been added into this figure incorrectly.

The authors were able to re-examine their original data, and have recognized how these errors occurred. The corrected versions of Fig. 2 and Fig. 4, now showing the correct data for the ABCB1 and ABCC5 experiments with the HCT116 cell line in Fig. 2B and the intended α-tubulin control bands in Fig. 4, are shown on the next two pages. Note that the errors made in assembling the data in these figures did not greatly affect either the results or the conclusions reported in this paper, and all the authors agree to the publication of this corrigendum. The authors regret that these errors went unnoticed prior to the publication of their article, are grateful to the Editor of *Oncology Letters* for granting them this opportunity to publish a corrigendum, and apologize to the readership for any inconvenience caused.

## Figures and Tables

**Figure 2.**
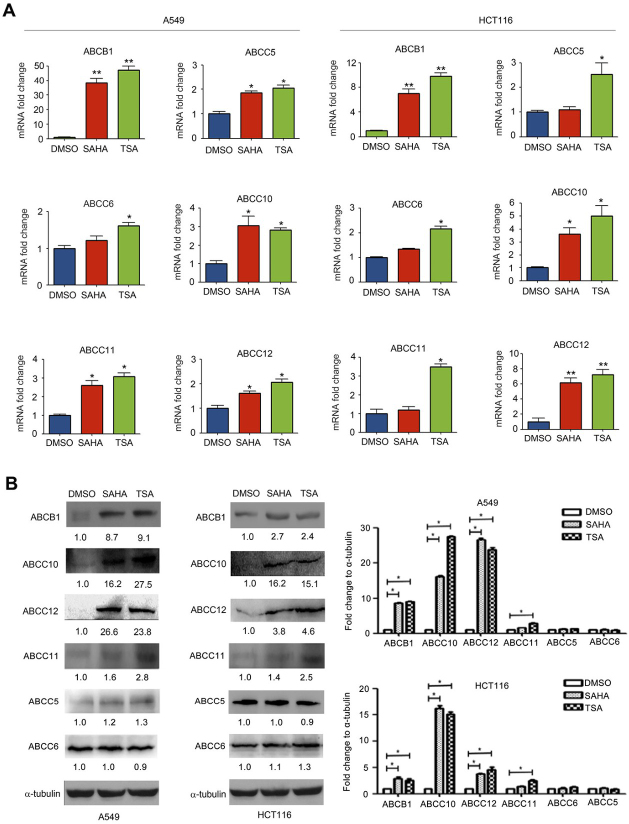
Histone deacetylase inhibitors increased the ABCB1, ABCC5, ABCC6, ABCC10, ABCC11 and ABCC12 expression levels. A549 and HCT116 cells were treated with DMSO, SAHA (0.5 µM) or TSA (100 nM) for 24 h. (A) mRNA expression levels of ABCB1, ABCC5, ABCC6, ABCC10, ABCC11 and ABCC12 were measured using reverse transcription-quantitative polymerase chain reaction. (B) Protein expression levels of ABCB1, ABCC5, ABCC6, ABCC10, ABCC11 and ABCC12 were detected using western blotting. *P<0.05 and **P<0.01, vs. DMSO group. ABC, ATP-binding cassette; SAHA, suberoylanilide hydroxamic acid; TSA, trichostatin A.

**Figure 4.**
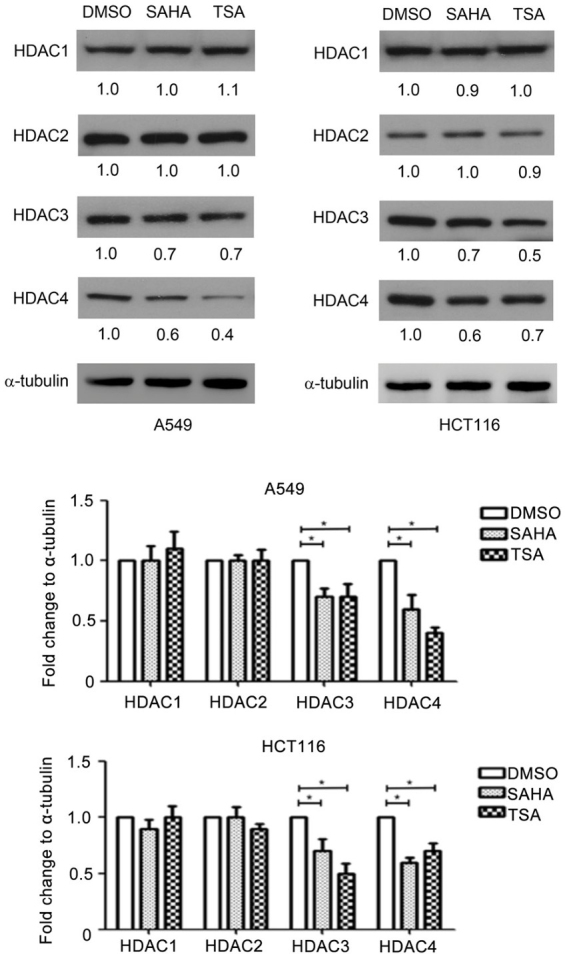
Effect of HDAC inhibitors on the HDAC1, HDAC2, HDAC3 and HDAC4 expression levels. A549 and HCT116 cells were treated with DMSO, SAHA (0.5 µM) or TSA (100 nM) for 24 h, following which HDAC1, HDAC2, HDAC3 and HDAC4 expression was detected using western blotting. *P<0.05 vs. DMSO group. HDAC, histone deacetylase; SAHA, suberoylanilide hydroxamic acid; TSA, trichostatin A.

